# Hepatic Fibrosis Is a Risk Factor for Greater Severity and Worse Outcome of Acute Ischemic Stroke

**DOI:** 10.3390/jcm11175141

**Published:** 2022-08-31

**Authors:** Eleftheria Ztriva, Adonis Protopapas, Pavlos Mentizis, Anastasios Papadopoulos, Christiana Gogou, Maria Kiosi, Maria Kyziroglou, Ioanna Minopoulou, Anastasia Gkounta, Erofili Papathanasiou, Evangelos Cholongitas, Christos Savopoulos, Konstantinos Tziomalos

**Affiliations:** 1 First Propedeutic Department of Internal Medicine, Medical School, Aristotle University of Thessaloniki, AHEPA Hospital, Thessaloniki 54636, Greece; 2 First Department of Internal Medicine, Medical School, National and Kapodistrian University of Athens, Laiko General Hospital, Athens 11527, Greece

**Keywords:** nonalcoholic fatty liver disease, nonalcoholic steatohepatitis, fibrosis, ischemic stroke, severity, outcome

## Abstract

Background: Nonalcoholic fatty liver disease, particularly in the presence of hepatic fibrosis, is associated with an increased risk of cardiovascular events, including ischemic stroke. However, it is unclear whether hepatic fibrosis is associated with the severity and outcome of acute ischemic stroke. Aim: To evaluate the relationship between hepatic fibrosis and the severity at admission and in-hospital outcome of acute ischemic stroke. Patients and methods: We prospectively studied all patients who were admitted to our department with acute ischemic stroke between September 2010 and February 2018 (*n* = 1107; 42.1% males, age 79.8 ± 7.2 years). The severity of stroke was assessed at admission with the National Institutes of Health Stroke Scale (NIHSS). Severe stroke was defined as NIHSS ≥ 21. The presence of hepatic fibrosis was evaluated with the Fibrosis-4 index (FIB-4). The outcome was assessed with dependency at discharge (modified Rankin Scale between 2 and 5) and with in-hospital mortality. Results: Patients with severe stroke had a higher FIB-4 index than patients with non-severe stroke (2.7 ± 1.7 and 2.3 ± 1.4, respectively; *p* < 0.05). Independent risk factors for severe IS were age (relative risk (RR) 1.064, 95% confidence interval (CI) 1.030–1.100, *p* < 0.001), female sex (RR 1.723, 95% CI 1.100–2.698, *p* = 0.012), atrial fibrillation (RR 1.869, 95% CI 1.234–2.831, *p* = 0.002), diastolic blood pressure (DBP) (RR 1.019, 95% CI 1.006–1.033, *p* = 0.001), and the FIB-4 index (RR 1.130, 95% CI 1.007–1.268, *p* = 0.022). At discharge, 64.2% of patients were dependent. The FIB-4 index did not differ between patients who were dependent and those who were independent at the time of discharge (2.3 ± 1.5 and 2.1 ± 1.2, respectively; *p* = 0.061). During hospitalization, 9.8% of patients died. Patients who died during hospitalization had a higher FIB-4 index than those who were discharged (2.9 ± 1.8 and 2.3 ± 1.4, respectively; *p* < 0.005). Independent risk factors for in-hospital mortality were DBP (RR 1.022, 95% CI 1.010–1.034, *p* < 0.001), serum glucose levels (RR 1.004, 95% CI 1.001–1.007, *p* = 0.007), serum triglyceride levels (RR 0.993, 95% CI 0.987–0.999, *p* = 0.023), NIHSS (RR 1.120, 95% CI 1.092–1.149, *p* < 0.001), and the FIB-4 index (RR 1.169, 95% CI 1.060–1.289, *p* = 0.002). Conclusions: Hepatic fibrosis, evaluated with the FIB-4 index, appears to be associated with more severe ischemic stroke and might also represent an independent risk factor for in-hospital mortality in patients admitted with acute ischemic stroke.

## 1. Introduction

Nonalcoholic fatty liver disease (NAFLD) is the most prevalent chronic liver disease worldwide [1,2,3]. NAFLD and atherosclerosis share many risk factors, including diabetes mellitus (DM), obesity, and insulin resistance [4,5]. Accordingly, several studies showed that NAFLD is related to higher risk of cardiovascular events [6,7,8,9]. Importantly, patients with hepatic fibrosis due to NAFLD have a greater cardiovascular risk, whereas isolated hepatic steatosis does not appear to increase the incidence of cardiovascular events [6,7,8,9].

Ischemic stroke (IS) is a major cause of mortality and long-term disability worldwide [10]. Several studies have shown that NAFLD, particularly in the presence of hepatic fibrosis, is an independent risk factor for IS [11,12,13,14,15]. Accumulating data suggest that hepatic fibrosis stimulates oxidative stress, induces inflammation, and activates the coagulation cascade, which in turn are implicated in the pathogenesis of IS [16]. However, there is limited data regarding the association between hepatic fibrosis and the severity and outcome of acute IS [17,18,19].

The aim of the present study was to evaluate the relationship between hepatic fibrosis and the severity and in-hospital outcome of acute IS. The FIB-4 index was used to evaluate the presence of hepatic fibrosis because it is non-invasive and inexpensive. However, it should be emphasized that this index requires prior diagnosis of liver disease and can also increase the risk of other liver diseases, e.g., liver metastases [20].

## 2. Patients and Methods

We prospectively studied all patients who were admitted to our department with acute IS between September 2010 and February 2018 (*n* = 1107; 42.1% males, age 79.8 ± 7.2 years). Methods have been described previously [21]. Briefly, demographic data and the presence of cardiovascular risk factors or established cardiovascular disease (CVD) were recorded. Anthropometric parameters and vital signs were measured at admission. Stroke severity was evaluated at admission with the National Institutes of Health Stroke Scale (NIHSS), and severe IS was defined as NIHSS ≥ 21 [22].

Routine laboratory investigations were performed at admission in the non-fasting status, including serum levels of alanine aminotransferase (ALT) and aspartate aminotrasnferase (AST). A brain computed tomography (CT) was performed in all patients at admission and was repeated if clinically indicated.

The presence of hepatic fibrosis was evaluated with the Fibrosis-4 index (FIB-4), which is calculated according to the following formula: [age × AST (IU/L)]/[platelets (×10^9^/L) × √ALT (IU/L)] [20].

All patients were tested for the presence of HBsAg and antiHCV, and those who were positive for either test were excluded from further analyses. Patients with excessive alcohol consumption were also excluded from the study. In addition, all patients who had elevated transaminase levels underwent liver ultrasound, which did not disclose primary hepatic cancer or liver metastases in any of these patients.

The outcome was assessed with dependency at discharge (i.e., modified Rankin Scale 2–5) and with in-hospital mortality.

The study was approved by the Ethics Committee of AHEPA Hospital, Thessaloniki, Greece. All patients provided written informed consent.

## 3. Statistical Analysis

IBM SPSS Statistics for Windows (version 27: Armonk, NY, USA: IBM Corp) was used for statistical analysis. Differences in categorical variables between groups were evaluated with the chi-square test. Differences in continuous variables between groups were evaluated with the independent samples *t*-test. Binary logistic regression analysis was used to identify independent predictors of severe IS and Cox regression analysis was used to identify independent predictors of longitudinal outcomes, i.e., dependency at discharge and in-hospital mortality. Variables that differed in univariate analyses between groups (i.e., between patients with severe and non-severe IS, between patients who were dependent and independent at discharge, and between patients who died during hospitalization and those who were discharged) were included in the regression models. FIB-4 was inserted as a continuous variable in all regression models.

## 4. Results

Patients’ characteristics stratified according to IS severity are shown in Table 1. At admission, 12.5% of patients had severe IS. Patients with severe IS had a higher FIB-4 index than those with non-severe stroke (2.7 ± 1.7 and 2.3 ± 1.4, respectively; *p* < 0.05). Other differences between the two groups are shown in Table 1. Independent risk factors for severe IS were age (relative risk (RR) 1.064, 95% confidence interval (CI) 1.030–1.100, *p* < 0.001), female sex (RR 1.723, 95% CI 1.100–2.698, *p* = 0.012), atrial fibrillation (AF) (RR 1.869, 95% CI 1.234–2.831, *p* = 0.002), diastolic blood pressure (DBP) (RR 1.019, 95% CI 1.006–1.033, *p* = 0.001), and the FIB-4 index (RR 1.130, 95% CI 1.007–1.268, *p* = 0.022).

At discharge, 64.2% of patients were dependent. The FIB-4 index was similar in patients who were dependent at discharge and those who were independent (2.3 ± 1.5 and 2.1 ± 1.2, respectively; *p* = 0.061). Differences between the 2 groups are shown in Table 2. Independent risk factors for dependency were age (RR 1.021, 95% CI 1.007–1.035, *p* = 0.004), prior IS (RR 1.251, 95% CI 1.042–1.502, *p* = 0.016), and NIHSS (RR 1.018, 95% CI 1.006–1.030, *p* = 0.004).

During hospitalization, 9.8% of patients died. Patients who died during hospitalization had a higher FIB-4 index than those who were discharged (2.9 ± 1.8 and 2.3 ± 1.4, respectively; *p* < 0.005). Other differences between the two groups are shown in Table 3. Independent risk factors for in-hospital mortality were DBP (RR 1.022, 95% CI 1.010–1.034, *p* < 0.001), serum glucose levels (RR 1.004, 95% CI 1.001–1.007, *p* = 0.007), serum triglyceride levels (RR 0.993, 95% CI 0.987–0.999, *p* = 0.023), NIHSS (RR 1.120, 95% CI 1.092–1.149, *p* < 0.001), and the FIB-4 index (RR 1.169, 95% CI 1.060–1.289, *p* = 0.002).

## 5. Discussion

In the present study, the FIB-4 index was independently associated with more severe acute IS. There is a paucity of data about the relationship between hepatic fibrosis and the severity of IS. In a small retrospective study from China (*n* = 384), patients with acute IS and higher FIB-4 had a higher NIHSS score at admission, but multivariate analysis was not performed to assess whether hepatic fibrosis is independently associated with IS severity [23]. In contrast, in a small retrospective study performed in Korea (*n* = 321), hepatic fibrosis evaluated with transient elastography was not associated with IS severity at admission [17]. Other studies reported that patients with elevated aminotransferase levels, presumably due to NAFLD, suffer more severe IS but did not evaluate the presence of hepatic fibrosis [24,25]. It is well-established that hepatic fibrosis is associated with cardiovascular morbidity [6,7,8,9]. This association appears to be partly due to the clustering of established cardiovascular risk factors–particularly obesity and type 2 diabetes mellitus–in patients with hepatic fibrosis but also due to the hepatic fibrosis-induced propagation of inflammation, thrombosis, and oxidative stress [16]. The findings of the present study suggest that patients with hepatic fibrosis are also at risk for more severe IS, which in turn is associated with greater mortality and disability [26]. On the other hand, it should be emphasized that we assessed the presence of hepatic fibrosis with the FIB-4 index, which is a surrogate marker of fibrosis and less accurate than liver biopsy [20]. Accordingly, our results should be interpreted with caution and require validation.

We did not find a relationship between hepatic fibrosis and the functional outcome at discharge of patients admitted with acute IS. Previous studies reported discordant findings regarding this association. In a small retrospective study in Caucasian patients with anterior circulation large vessel occlusion IS who underwent mechanical thrombectomy (*n* = 460), FIB-4 was an independent predictor of functional outcome at 3 months post-stroke [18]. In contrast, in a small retrospective study in Korean patients (*n* = 321), hepatic fibrosis evaluated with transient elastography was not associated with the functional outcome at 3 months after IS [17]. In two other studies (*n* = 200 and 306, respectively), patients with acute IS and elevated aminotransferase levels presumably due to NAFLD had worse functional outcome at discharge in the first [24], but no such relationship was observed in the other [25]. However, neither of these studies evaluated the presence of hepatic fibrosis [24,25]. Given the strong relationship between the severity of IS and the functional outcome, it is possible that negative studies lacked power to identify a relationship between hepatic fibrosis and dependency [17,24,25]. Indeed, in our study, we observed a trend for a higher FIB-4 index in patients who were dependent at discharge (*p* = 0.061).

Another potentially important finding of our study is that FIB-4 predicted in-hospital mortality. In a recent report, FIB-4 was an independent predictor of mortality at 3 months post-stroke in a cohort of 460 Caucasian patients with anterior circulation large vessel occlusion IS who underwent mechanical thrombectomy [18]. In two studies from the same group performed in Korea, hepatic fibrosis evaluated with the FIB-4 index or with transient elastography predicted all-cause and cardiovascular mortality after a median follow-up of 1.2 and 2.7 years, respectively [19,27]. Neither of these studies evaluated the impact of hepatic fibrosis on in-hospital mortality [19,27]. It is of interest that the association between hepatic fibrosis and mortality was independent of IS severity and other traditional risk factors for adverse outcome in this population, including atrial fibrillation and elevated BP [26]. It is possible that subclinical inflammation, oxidative stress, and a procoagulant state, which accompany nonalcoholic steatohepatitis [4,5], might contribute to the higher mortality of patients with hepatic fibrosis who suffer an acute IS.

In conclusion, the present study suggests that hepatic fibrosis, evaluated with the FIB-4 index, is associated with more severe IS and is also an independent risk factor for in-hospital mortality in patients admitted with acute IS. Given that the FIB-4 index is easily calculated using readily available, inexpensive, and routine laboratory parameters, it might represent a useful tool for the timely identification and aggressive management of patients at increased risk for these adverse outcomes.

## Figures and Tables

**Table 1:** Patients’ characteristics at admission stratified according to stroke severity (*n* = 1107).

	Patients with Severe Stroke (*n* = 138)	Patients with Non-Severe Stroke (*n* = 969)	*p*
Age (years)	82.7 ± 7.5	79.5 ± 7.0	<0.001
Males (%)	30.4	44.1	<0.005
Systolic blood pressure (mmHg)	152 ± 28	150 ± 26	0.299
Diastolic blood pressure (mmHg)	84 ± 17	80 ± 14	0.006
Heart rate	81 ± 17	78 ± 15	0.080
Hypertension (%)	79.7	80.6	0.976
Type 2 diabetes mellitus (%)	33.3	32.6	0.899
Type 2 diabetes mellitus duration (years)	13.6 ± 8.7	12.2 ± 8.7	0.404
Smoking (current/past, %)	10.1/7.9	14.9/15.3	0.020
Package-years	76 ± 53	45 ± 18	0.109
Atrial fibrillation (%)	54.3	34.6	<0.001
Body mass index (kg/m^2^)	27.7 ± 6.2	27.6 ± 4.8	0.884
Waist circumference (cm)	104 ± 11	101 ± 11	0.248
Waist/hip	0.98 ± 0.07	1.07 ± 0.09	0.064
Overweight/obese (%)	29.7/29.7	41.4/28.1	0.070
Family history of cardiovascular disease (%)	12.3	19.2	0.068
Coronary heart disease (%)	25.4	25.3	1.000
Prior ischemic stroke (%)	43.5	48.5	0.315
Heart failure (%)	21.0	14.9	0.123
Glucose (mg/dL)	138 ± 65	113 ± 46	0.061
Low-density lipoprotein cholesterol (mg/dL)	107 ± 35	107 ± 39	0.903
High-density lipoprotein cholesterol (mg/dL)	50 ± 18	47 ± 14	0.081
Triglycerides (mg/dL)	102 ± 56	118 ± 56	0.073
Uric acid (mg/dL)	5.9 ± 2.2	5.7 ± 1.8	0.300
Estimated glomerular filtration rate (mL/min/1.73 m^2^)	62 ± 20	64 ± 20	0.274
National Institutes of Health Stroke Scale	27.3 ± 5.2	6.3 ± 5.5	<0.001
Fibrosis-4 index	2.7 ± 1.7	2.3 ± 1.4	0.010

**Table 2:** Significant differences between patients who were dependent at discharge (modified Rankin Scale 2–5) and those who were independent at discharge (modified Rankin Scale 0–1).

	Patients Dependent at Discharge (*n* = 711)	Patients Independent at Discharge (*n* = 396)	*p*
Age (years)	80.6 ± 7.3	77.3 ± 6.5	<0.001
Male sex (%)	37.8	47.2	<0.01
Heart rate	79 ± 15	76 ± 14	<0.01
Smoking (current/past, %)	13.6/12.1	14.4/20.4	<0.005
Atrial fibrillation (%)	37.8	27.0	<0.001
Family history of cardiovascular disease (%)	21.1	12.9	<0.005
Prior ischemic stroke (%)	53.7	35.3	<0.001
Glucose (mg/dL)	116 ± 48	107 ± 39	<0.005
Estimated glomerular filtration rate (mL/min/1.73 m^2^)	62 ± 20	67 ± 19	<0.001
National Institutes of Health Stroke Scale	9.8 ± 7.3	2.4 ± 2.7	<0.001

**Table 3:** Significant differences between patients who died during hospitalization and those who were discharged.

	Patients Who Died during Hospitalization (*n* = 108)	Patients Who Were Discharged (*n* = 999)	*p*
Age (years)	83.0 ± 7.1	79.5 ± 7.1	<0.001
Diastolic blood pressure (mmHg)	88 ± 18	80 ± 14	<0.001
Heart rate	83 ± 16	78 ± 15	<0.001
Atrial fibrillation (%)	60.2	33.8	<0.001
Glucose (mg/dL)	149 ± 75	113 ± 45	<0.001
Low-density lipoprotein cholesterol (mg/dL)	98 ± 38	108 ± 38	<0.05
Triglycerides (mg/dL)	98 ± 54	118 ± 56	<0.005
Uric acid (mg/dL)	6.2 ± 2.0	5.7 ± 1.9	<0.05
National Institutes of Health Stroke Scale	23.8 ± 8.3	7.3 ± 7.2	<0.001
Fibrosis-4 index	2.9 ± 1.8	2.3 ± 1.4	<0.005
